# The effects of early rehabilitation on functional exercise tolerance in decompensated heart failure patients: Results of a multicenter randomized controlled trial (ERIC-HF study)

**DOI:** 10.1177/02692155221088684

**Published:** 2022-03-21

**Authors:** Bruno Delgado, André Novo, Ivo Lopes, Carina Rebelo, Cecília Almeida, Sandra Pestana, Bárbara Gomes, Erika Froelicher, Leonie Klompstra

**Affiliations:** 1 Centro Hospitalar Universitário do Porto, Instituto de Ciências Biomédicas Abel Salazar; 2 386399 Instituto Politécnico de Bragança, CINTESIS:NursID, Portugal; 3 Centro Hospitalar Universitário do Porto; 4 Hospital Infante D. Pedro; 5 230250 Centro Hospitalar De Setúbal; 6 59043 Centro Hospitalar de Vila Nova de Gaia/Espinho; 7 112123 Escola Superior de Enfermagem do Porto, CINTESIS:NursID, Portugal; 8 Department of Physiological Nursing, School of Nursing and Department of Epidemiology & Biostatistics, School of Medicine, University of California San Francisco, USA; 9 Departement of Social and Welfare Studies, Linköping University, Linköping, Sweden

**Keywords:** Functional exercise tolerance, activities of daily living, heart diseases, functional independence, heart failure

## Abstract

**Objective:**

To analyze (1) the effect of an aerobic training program on functional exercise tolerance in decompensated heart failure (DHF) patients; (2) to assess the effects of an aerobic training program on functional independence; and (3) dyspnea during activities of daily living.

**Design:**

A randomized controlled clinical trial with follow-up at discharge.

**Settings:**

Eight hospitals. Recruitment took place between 9/ 2017 and 3/2019.

**Group Assignments:**

Patients with DHF who were admitted to the hospital, were randomly assigned to usual rehabilitation care guideline recommended (control group) or aerobic training program (exercise group).

**Main outcome:**

Functional exercise tolerance was measured with a 6-min walking test at discharge.

**Results:**

In total 257 patients with DHF were included, with a mean age of 67 ± 11 years, 84% (*n* = 205) had a reduced ejection fraction and the hospital stay was 16 ± 10 days. At discharge, patients in the intervention group walked further compared to the control group (278 ± 117m vs 219 ± 115m, *p* < 0.01) and this difference stayed significant after correcting for confounders (*p* < 0.01). A significant difference was found favoring the exercise group in functional independence (96 ± 7 vs 93 ± 12, *p* = 0.02) and dyspnea associated to ADL (13 ± 5 vs 17 ± 7, *p* < 0.01) and these differences persisted after correcting for baseline values and confounders (functional independence *p* < 0.01; dyspnea associated with ADL *p* = 0.02).

**Conclusion:**

The ERIC-HF program is safe, feasible, and effective in increasing functional exercise tolerance and functional independence in hospitalized patients admitted due to DHF.

## Introduction

Decompensated Heart Failure (DHF) is characterized by the inability of the heart to eject and/or accommodate blood within physiological pressure levels; it represents approximately 60% of the total cost with heart failure (HF) treatment, responsible for almost 10% of mortality.^1^ Patients with DHF often experience symptoms like breathlessness and edema. Patients often have functional dependence, impairment of performance in activities of daily living (ADL) as well as limitations in social life and, consequently, decreased quality of life.^1–6^

Besides guideline-recommended drugs, the treatment of HF involves non-pharmacological interventions such as cardiac rehabilitation, where exercise training is one of the core components.^7^ Exercise training programs have shown to be a safe, economic and feasible therapeutic resources.^4,6^ Moreover, numerous benefits have been shown, such as an increase of functional exercise tolerance, quality of life, reduction of hospitalization readmissions and cardiovascular mortality.^6,8^ Because of these benefits, cardiac rehabilitation should be offered as soon as the patients is clinical stable and should continue throughout hospitalization.^4^

Unfortunately, guideline recommendations are imprecise in terms of the volume and duration of exercise.^9^ Until now, scarse data exists about patients with DHF in exercise training during the phase of stabilization.^10^ Early rehabilitation in patients with DHF was associated with a higher rates of recovery in ADL^11^ and improved sleep quality.^12^ A pilot study^13^ showed that aerobic training in patients with DHF was safe and feasible. Therefore, the aim of this trial is to test the effects of aerobic training on functional exercise tolerance of patients with DHF, on functional independence and dyspnea during ADL.

## Methods

This multicenter randomized controlled, study is called “Early Rehabilitation in Cardiology-Heart Failure (ERIC-HF)”. The trial was registered at clinicaltrials.gov (NCT03838003) and approved by the respective Ethics Committees (referee 172/17; 82943/17; 560/2018; 393/17; 17/2018; 58/18; and 244/18).

The trial protocol has been published in a previous report.^13^ Patients were recruited from eight cardiology wards (two university hospitals and six regional hospitals). The enrollment started at September 2017 and ended March 2019. All participants provided written consent following the principles outlined in the Declaration of Helsinki. This report is organized according to the CONSORT guidelines.

### Eligibility criteria

A cardiologist and a cardiovascular nurse for eligibility evaluated all patients admitted due to decompensation of HF. After the patient was medically stable, they were evaluated for the eligibility criteria. Patients older than 18 years with diagnosed of DHF (independent of the etiology or the systolic left ventricular function) were eligible to be included in the study. Patients unable to understand the exercises due to cognitive impairment or had osteoarticular impairment for walking were excluded.

### Randomization

The sample were randomized into the control group or the exercise group using an online randomization program.^14^ Stratified randomization by center were carried out to control for potential center-based confounders. For every center 30 randomizations were created in the online randomization program.

### Group assignment

*Control group:* All patients with DHF received the usual treatment according to the American College of Sports Medicine (ACSM) guidelines as well as information about cardiac rehabilitation and physical activity.^9^ The guidelines recommend walking short-to-moderate distances, with minimal or no assistance, three to four times a day, progressing to independent ambulation; upper body movement exercises and minimal stair climbing, but are unclear about the time and duration of it.^9^ In the ERIC-HF study, patients were encouraged to perform exercise according to these recommendations for a minimum of 5 to a maximum of 20 min a day.

*Exercise group:* In addition to usual care, patients in the exercise group performed an aerobic training program, that was composed by five progressive intensity stages and was adjusted for the individual capacity of each patient: Stage 1: Respiratory and callisthenic exercises performed in supine or orthostatic position; Stage 2: 5 to 10 min on cycle ergometer; Stage 3: 5 to 10 min walking; Stage 4: 10 to 15 min walking; Stage 5: 10 to 15 min walking and 5 min climbing stairs (patients may stop for recovery)

The program was designed according to the Frequency/Intensity/Type/Time-Volume/Progression (FITT-VP) parameters.^9^ The frequency was five days per week, twice a day during the hospital stay. Patients who were in New York Heart Association Functional Classification (NYHA-Class) III^6,15^ and were able to paddle could start in the second stage of the program, all other patients started in the first stage. If the patient was not able to perform the minimum duration of the stage, or if there was any decompensation or if they reported their perceived subjective effort as greater than eight, the patient would go back to the previous stage. A more detailed description of the aerobic training program is published previously.^13^

### Outcome assessment

*Functional exercise tolerance* was assessed at discharge by the six-minute walking test (6MWT) in accordance with the American Thoracic Society guidelines.^16,17^ The patients performed the walk at their usual speed as recommended. This is an easy-to-administer, inexpensive and safe test that assesses patient's submaximal functional exercise tolerance. This test is found to be valid and reliable.^18^

*Functional independence* levels during ADL were assessed when patients were admitted to the hospital and the day of discharge and was collected using a questionnaire. Functional independence was measured with the Barthel Index. This index assesses the level of independence of the person in performing 10 ADL: eating, personal hygiene, toilet use, bathing, dressing and undressing, sphincter control, walking, transferring from the chair to the bed and climbing stairs.^19,20^ The minimum score of 0 corresponds to the maximum dependency for all the ADL evaluated, and the maximum score of 100 equals the total independence for the same ADL.^21^ Index scores of 0–20 indicate “total” dependency, 21–60 indicate “severe” dependency, 61–90 indicate “moderate” dependency, and 91–99 indicates “slight” dependency.

*Dyspnea level* during ADL was assessed the day patients were admitted to the hospital and the day of discharge with the London Chest of Activities of Daily Living (LCADL). This instrument assessed the limitation that dyspnea causes in the performance of the ADL. It is a questionnaire of 15 items, divided into 4 domains (self-care, domestic care, leisure, and physical activity), with each item scored from 0 to 5, in a maximum of 75 points. The higher the value, the greater the limitation in ADL due to dyspnea.^22^

*Demographic and clinical data* were collected from the medical charts, e.g. age, gender, New York Heart Association Class (NYHA-Class), left ventricular ejection function (LVEF), etiology of HF, cardiovascular risk factors, adverse events.

### Data analyses

The Statulator^23^ sample size calculator was used for sample size calculation. An effect size was set of 40 meters standard deviation of 110.4 meters (derived from a pilot study,^13^^)^ the statistical power was set as 0.8 and the alpha as 0.05, yielded a sample size of 120 for each group to achieves. Assuming a 15% drop-out rate, 276 patients in total were needed.

Patient characteristics were reported as absolute (relative) frequency or means (±sd). Normal distribution was assessed with the Kolmogorov-Smirnov test Primary and secondary outcomes at the day of discharge were analyzed with an intention-to-treat analysis. The primary outcome was assessed with Linear regression, corrected for known prognostic factors for DHF (age, gender, NYHA-Class, left ventricular function and total time of in-hospital stay). Secondary outcomes were assessed by analysis of covariance (ANCOVA), with baseline measures, age, gender, NYHA-Class, left ventricular function and total time of in-hospital stay as covariates. A paired t-test using Bonferroni's correction between the control group and the exercise group was done as a post-hoc test

Missing data was not replaced. All statistics were performed using SPSS version 24.0.

## Results

**Figure 1.**
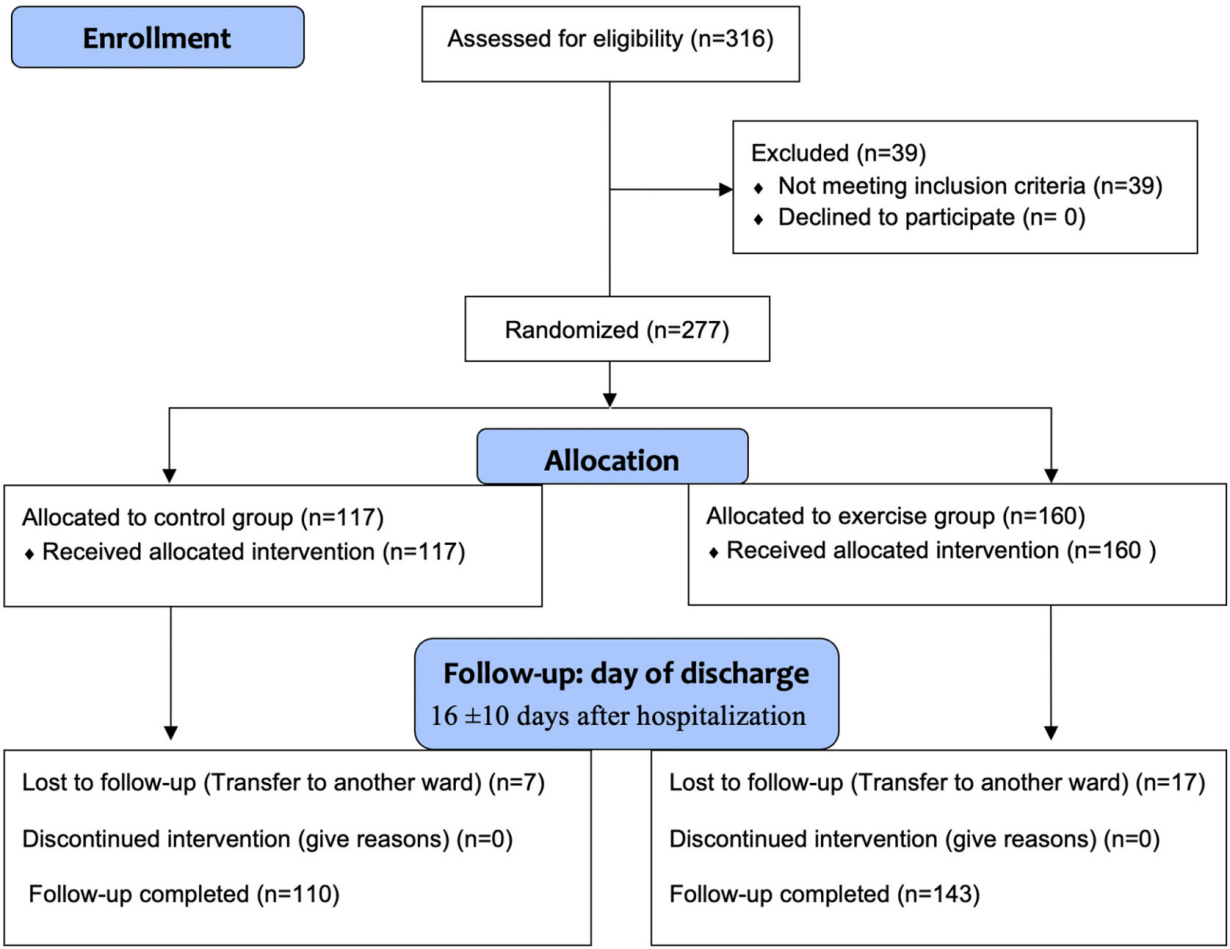
CONSORT flowchart.

Of the 316 patients who were assessed for eligibility, 39 did not made meet the inclusion criteria. In total 277 patients were included; 117 patients were allocated to the control group and 160 patients were allocated to the exercise group (Figure 1). At discharge, 24 patients were loss to follow-up (7 patients in the control group and 17 patients in the exercise group) due to their transfer to another ward in the hospital. No patients died during the study; no major adverse events were reported. Patient's mean age was 67 (±11) years old (66% male) (Table 1).

**Table 1.** Characteristics of patient with decompensated heart failure.

Parameter	Total (*n*** **=** **277)	Control group (*n*** **=** **117)	Exercise group (*n*** **=** **160)
Age (years)	68 ± 11	68 ± 11	67 ± 10
Female gender	33% (92)	36% (45)	32% (49)
In-hospital stay (days)	16 ± 10	16 ± 11	17 ± 9
**NYHA class**			
III	83% (232)	85% (99)	83% (133)
IV	17% (45)	15% (18)	17% (27)
**LVEF**			
HFrEF	79% (219)	77% (90)	80% (129)
HFmEF	6% (17)	10% (12)	3% (5)
HFpEF	15% (41)	13% (15)	17% (26)
**Aetiology**			
Valvular	36% (99)	33% (41)	36% (58)
Ischemic	31% (85)	32% (30)	35% (55)
Dilated	9% (25)	8% (17)	5% (8)
Other	24% (68)	27% (29)	25% (39)

HFmEF, Heart Failure mid-range Ejection Fraction; HFpEF, Heart Failure preserved Ejection Fraction; HFrEF, Heart Failure reduced Ejection Fraction; NYHA class, New York Heart Association Functional Classification.

The hospital stay was 16 (±10) days. An average of 12 (±5) training sessions was performed. Patients were not always able to perform the aerobic training program twice a day according to study protocol due to clinical concerns, namely the need to carry out diagnostic or therapeutic exams. In total, 23% of patients (*n* = 36) were able to attend all the training sessions, 42% (*n* = 67) were able to compose 80% or more of the training sessions, and 24% (*n* = 39) were able to attend 59% or more of the training sessions.

### Primary end-point (functional exercise tolerance)

At the day of discharge, patients walked on average 252 (± 120) meters on the 6MWT (Table 2). A significant difference between the control group and the exercise group was found in the distance walked on the six-minute walk test in favor of the exercise of 59 meters. This difference remained significant when controlling for age, gender, NYHA-Class, left ventricular function and total time of hospital stay (*p*-value<0.01) (Table 3).

**Table 2.** Univariate analyses on the difference between exercise group and control group in 6-min walk test, barthel Index, LCADL at the day of discharge.

**Values at discharge**	**Total** (***n* **=** **253)	**Exercise group** (***n* **=** **143)	**Control group** (***n* **=** **110)	**P-Value**
6-min walk test	252 ± 120	278 ± 117	219 ± 115	<.001
Barthel Index	94 ± 9	96 ± 7	93 ± 12	0.03
LCADL	14 ± 6	13 ± 5	17 ± 7	<.001

**Table 3.** Difference between the exercise group and control group in functional exercise tolerance at discharge, controlled for possible confounding factors (age, gender, NYHA-class, LVEF, In-hospital stay).

	**Standardized *β***	**95% CI**	***P*** **-value**	***R*** **^2^**	***F***
Functional exercise tolerance*				0.17	5.11
** Randomization (1 exercise group; 2 control group)**	−44.31	−83.83 – −4.79	**0.03**		
** Age (years)**	−3.43	−5.17 – −1.69	**<.001**		
** Gender (1 male, 2 female)**	−56.39	−101.50 – −11.28	**0.02**		
** NYHA**	−14.36	−77.27–48.54	0.65		
** LVEF**	−4.86	−23.63–13.92	0.61		
** In-hospital stay (days)**	−1.24	−3.95–1.46	0.36		

* Measured with the 6-min walking test LVEF, Left Ventricular Ejection Fraction; NYHA-Class, New York Heart Association Classification.

Bold values are statistically significant.

### Functional independence

At hospitalization, the Barthel Index score was on average 75 (±18). At the day of discharge, a statistically difference in functional independence was found favoring the exrecise group (mean control group 93 (±12) vs mean exercise group 96 (±7), *p*-value = 0.03) (Table 2). This difference stayed significant when controlling for baseline (*p*-value<0.002), but was not significant when controlling for baseline and confounding factors (*p* = 0.11) (Table 4).

**Table 4.** Difference between the exercise group and control group in functional independence and dyspnea level during activity of daily living over time (baseline and at discharge) and controlled for possible covariates (age, gender, NYHA-class, LVEF, In-hospital stay).

Outcomes	With corrections for baseline				With corrections for baseline and possible covariates			
** **	95% CI	P-Value	*R* ^2^	*F*	95% CI	P-Value	*R* ^2^	*F*
			0.35	67.23			0.55	19.45
Level of independence*	[−5 to −1.1]	**0.002**			[−5 to −0,47]	0.105		
			0.32	57.92			0.47	14.22
Dyspnea level during activity**	[2.2 to 4.8]	**<.001**			[0.9 to 3.3]	**<.001**		

* Measured with the Barthel Index.

** Measured LCADL, London Chest Activities of Daily Living scale.

LVEF, Left Ventricular Ejection Fraction; NYHA-Class, New York Heart Association Classification.

Bold values are statistically significant.

### Dyspnea level

At hospitalization, the LCADL score was on average 31 (±8) (Table 2). At the day of discharge, a difference was found in dyspnea level during ADL, favoring the IG (17 ± 7 CG vs mean for IG 13 (±5), *p*-value<0.001) (Table 2). This difference stayed significant when controlling for baseline (p-value<0.001) and baseline and confounding factors (*p*-value<0.001) (Table 4).

## Discussion

The findings of our study show that aerobic training is effective in patients with DHF to increase functional exercise tolerance. A 59 meters difference on the 6MWT was found in favor of the exercise group, which is considered clinically relevant. Other studies showed that a difference of 12–35 meters on the 6MWT is clinically important in patients with heart failure.^24–26^ The 6MWT is proved to be useful as an outcome in clinical trials, as a decline in the 6MWT was highly associated with 1-year mortality. The clinically relevant change in 6MWT that was found in our study is remarkable, considering that our study included patients with DHF. One reason could be the type of exercise chosen in this study. Other studies report that up to a third of patients that completed cardiac rehabilitation did not improve their functional exercise tolerance due to exercise training performed at too low of an intensity^27^ or due to chronotropic incompetence.^28^ In our study we offered patients aerobic training, and this training form and high-intensive interval training, but not resistance training, are associated with increased telomerase activity and telomere length in mononuclear cells.^29^

Patients who participated in aerobic training also had benefits in functional independence and dyspnea level during ADL, demonstrating that patients performed aerobic training program can achieve a greater improvement on their functional exercise tolerance. In a systematic review regarding the effects of exercise on functional status of acutely hospitalized older adults, studies demonstrate that the performance of supervised exercise training is of great importance for the improvement or even attenuation of the functional impairment that hospitalization can bring.^30^ A systematic review identified physical function as a risk factor associated with adverse outcomes in elderly patients after hospital discharge.^31,32^ Thus, the assessment of physical functions, functional independence and dyspnea level during ADL in patients with DHF, are important since they can be used to determine prognosis or prevent functional impairment.^33,34^

The European Society of Cardiology Clinical Practice Guidelines for Acute and Chronic Heart Failure Guidelines recommends that patients with HF, regardless of LVEF to perform properly designed exercise training.^6^ Unfortunately, there is limited data on the safety and clinical outcomes related to exercise training in patients with DHF.^35^ These patients have often been excluded from previous exercise interventions. Aerobic training showed not only effective to increase functional exercise tolerance, but in our sample, it seemed safe, and the intervention was feasible to include hospitalized patients with DHF and possible to implement in different settings (cardiology wards in university hospitals and regional hospitals).

The present study has some limitations. The follow-up measurements were performed the day of discharge from the hospital and no longer follow-up was performed. Patients included had different lengths of hospital stay, therefore the number of exercise sessions between patients differed. We found an imbalance in numbers of patients between the control group and the exercise group. This imbalance was found at every center. This imbalance was due to that in every center 30 randomization were prepared in the online randomization program and most centers did not include 30 patients. In future studies, greater supervision of the process will be needed, and smaller blocks in the randomization should be used.

**Clinical messages**

- The salient findings of our study lead to some key messages to clinical practitioners, namely:
- Aerobic training can improve exercise and functional exercise tolerance in patients with DHF.
- Aerobic training appears to be safe and feasible in patients with DHF during hospitalization
